# Management of Psoriasis Herpeticum in Pregnancy: A Clinical Conundrum

**DOI:** 10.1155/2016/5319425

**Published:** 2016-10-20

**Authors:** Leanne Almario, Albert S. Antonyan, Dennis A. Porto, Hunter Gomez-Roberts, Ali Alhousseini, Bernard Gonik

**Affiliations:** ^1^Department of Obstetrics and Gynecology, Detroit Medical Center, Wayne State University, 3990 John R., 7 Brush N., Detroit, MI 48201, USA; ^2^Department of Dermatology, Henry Ford Health System, 3031 West Grand Boulevard, Suite 800, Detroit, MI 48202, USA

## Abstract

*Introduction.* Kaposi varicelliform eruption (KVE) is a widespread cutaneous viral infection, most commonly herpes simplex virus, which affects patients with underlying dermatosis. When KVE occurs in a patient with a history of psoriasis, it is referred to as psoriasis herpeticum, a rare subtype of KVE with only a handful of cases reported in the literature. To the authors' knowledge, we report for the first time a case of psoriasis herpeticum in pregnancy.* Case Presentation.* A 23-year-old woman in her third pregnancy presented at 26-week gestation with a 10-year history of psoriasis. Cutaneous examination revealed diffuse psoriatic plaques with scattered ~1 cm erosions. Punch biopsy of the skin revealed herpes simplex virus (HSV) infection within a psoriatic plaque, necessitating dermatological treatment. The patient experienced premature rupture of membranes at 37-week gestation. Pelvic exam showed no evidence of herpetic lesions. After labor augmentation, the patient delivered a healthy female infant with no evidence of HSV infection.* Discussion.* Psoriasis herpeticum is a rare and potentially devastating complication of an underlying dermatosis. With a paucity of data available to guide pregnancy-specific issues, the general management of this condition is controversial and requires a multidisciplinary care approach. Concerns for systemic infection in the mother and vertical transmission to the neonate are of critical importance.

## 1. Introduction

Kaposi varicelliform eruption (KVE) is a widespread cutaneous infection that occurs in patients with an underlying dermatosis [1]. Several viruses including herpes simplex virus (HSV) type 1 and type 2 and, more rarely, small pox virus and coxsackievirus A16 have been associated with KVE. When KVE occurs in a patient with a known history of psoriasis, it is referred to as psoriasis herpeticum [1]. Psoriasis herpeticum is a rare subtype of KVE with only a handful of cases reported in the literature. To the authors' knowledge, after performing a literature search utilizing the terms “Kaposi varicelliform eruption,” “psoriasis herpeticum,” “eczema herpeticum,” and “pregnancy” in PubMed, we report for the first time a case of psoriasis herpeticum in a pregnant patient.

## 2. Case Presentation

A 23-year-old African American woman in her third pregnancy presented at 26-week gestation for prenatal care. She reported a 10-year history of psoriasis, which flared with her prior pregnancy but was otherwise well controlled with topical corticosteroids. She described worsening of her psoriatic lesions with the current pregnancy, with an increase in the number of burning, pruritic, scaly plaques to which she had only been applying white petrolatum. She also described new flaccid blisters overlying her psoriatic plaques with this recent flare. She denied any joint pain or other systemic symptoms and otherwise felt well. Both she and her partner denied a history of herpes simplex virus or other sexually transmitted infections.

**Figure 1:**
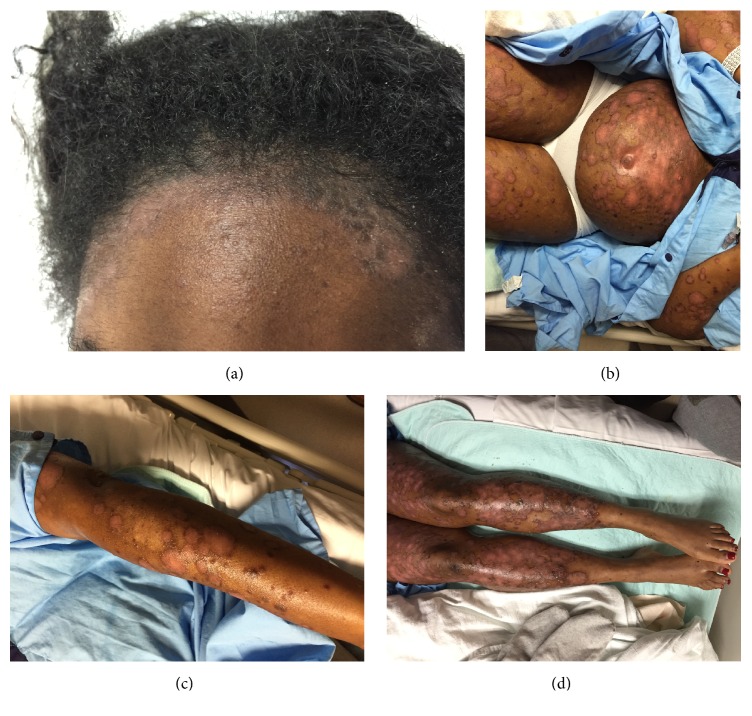
Psoriasis herpeticum. Erythematous scaly plaques with peripheral hyperpigmentation involving the (a) face, scalp, (b–d) torso, and extremities, with scattered ~1 cm erosions.

On examination, she was in no acute distress, and vital signs were stable and within normal limits. Cutaneous examination revealed erythematous scaly plaques with peripheral hyperpigmentation involving the scalp, face, arms, abdomen including the umbilicus, and pretibial legs, with scattered ~1 cm erosions within the plaques (Figure 1).

**Figure 2:**
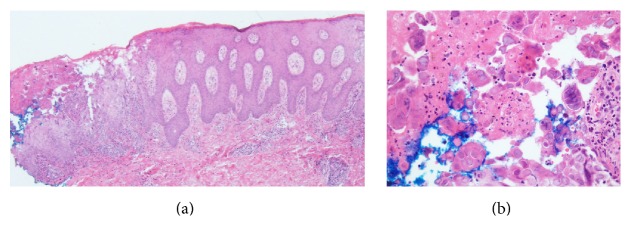
Psoriasis herpeticum. Punch biopsy specimen of the left leg demonstrating (a) psoriasiform acanthosis, epidermal necrosis, and acantholysis with (b) multinucleate keratinocytes with margination of chromatin and nuclear molding (hematoxylin-eosin stain; original magnification: (a) ×40; (b) ×400).

**Figure 3:**
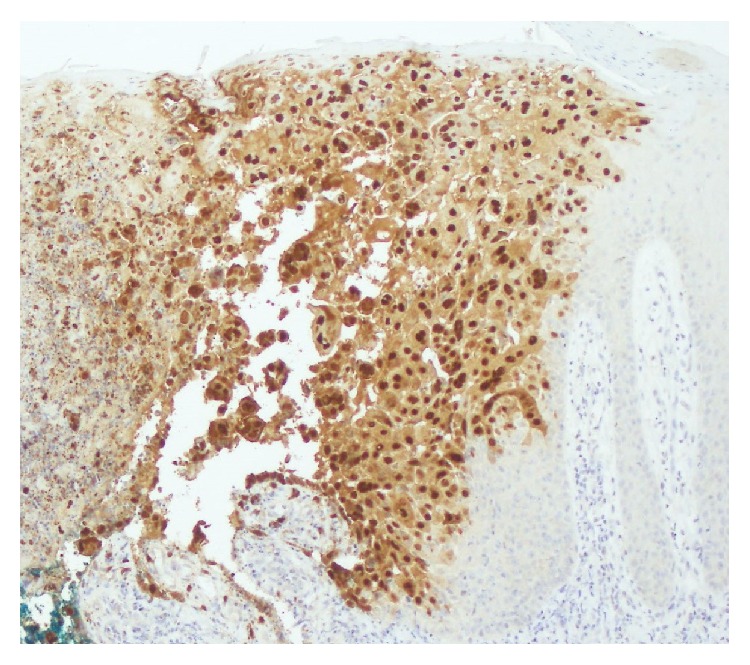
Psoriasis herpeticum. Biopsy specimen from Figure 2 showing positivity with HSV type I immunoperoxidase staining (herpes simplex virus type I immunoperoxidase stain; original magnification: ×200).

Punch biopsy of a representative skin lesion revealed psoriasiform acanthosis, epidermal necrosis, and acantholysis, as well as multinucleate keratinocytes with margination of chromatin and nuclear molding (Figure 2). Immunoperoxidase staining by monoclonal antibody for HSV type I and type II was positive, in keeping with the frequent cross-reactivity encountered with these antibody preparations (Figure 3). Direct immunofluorescence of perilesional skin was negative. These histologic and immunohistochemical findings were consistent with a diagnosis of herpes simplex infection within psoriatic plaques. HSV serology was positive for HSV IgM. Her HSV-1 IgG titer was negative and HSV-2 IgG was positive. HSV type 1 and 2 viral cultures obtained from the cervix were reported as negative.

She was started on a 14-day course of valacyclovir therapy and then switched to a daily prophylaxis regimen, with improvement of the blistering component of her skin lesions. She underwent a 1-week course of outpatient daily triamcinolone ointment wraps and narrow-band UVB phototherapy, as well as daily topical corticosteroids, with improvement of her cutaneous psoriasis.

At a follow-up prenatal visit, the patient complained of generalized itching and was switched to acyclovir prophylaxis. At 35-week and 4-day gestation, the patient self-discontinued antiviral therapy due to continued generalized itching, resulting in recurrence of the intralesional herpetiform vesicles. The Dermatology Service was again consulted for treatment recommendations of erythroderma secondary to psoriasis flare with superimposed HSV infection. The patient was treated with daily inpatient triamcinolone wet wraps until she was no longer erythrodermic. Oral antihistamine therapy was initiated to improve acyclovir tolerability related to her reported pruritus.

At 37-week and 1-day gestation, the patient presented to labor and delivery with premature rupture of membranes. Upon admission, no cutaneous or mucosal HSV lesions were noted. Labor was augmented with oxytocin. The patient delivered a healthy female infant without any observable HSV lesions. As such, neonatal HSV cultures were not obtained and prophylactic antiviral therapy was not initiated. At the infant's one-week pediatric visit, the baby was noted to be doing well.

## 3. Discussion

Kaposi varicelliform eruption was first described and reported by Moritz Kaposi in 1887 [2]. Since its introduction, a clear pathogenesis for KVE has yet to be defined. It is speculated that a combination of impaired barrier function and a defective host immune response makes individuals more vulnerable to KVE [3]. Different nomenclatures have been utilized to describe the different types of KVE based on either the causative virus or the preexisting dermatosis [3]. While the most common preexisting dermatosis is atopic dermatitis, a number of other dermatoses have also been implicated in KVE, including psoriasis [3]. While several cases of KVE in pregnancy have been reported in the literature [4–9], to our knowledge, we report the first documented case of psoriasis herpeticum in the setting of pregnancy.

Worldwide, the adult serologic positivity rate for HSV infection, including both HSV-1 and HSV-2, approaches 90% [10]. Although many individuals infected with HSV report oral or genital lesions, the majority are either asymptomatic or undiagnosed. In the United States, 1 in 6 adults are seropositive for HSV-2, with the highest prevalence seen in the African American female population [11, 12]. The majority of these cases are thought to be genital in nature. This family of DNA viruses can be associated with both primary and recurrent disease. It is not uncommon for patients to be unaware of a prior history of HSV infection, and it is often difficult to decipher primary versus recurrent disease with certainty. HSV IgM positivity can be seen with primary or recurrent disease, while HSV IgG positivity typically refers to a prior history of infection with or without reactivation. Despite the typical finding of recurrent HSV disease following an isolated dermatomal pattern, in the setting of a diffuse dermatologic condition such as psoriasis, spread across multiple dermatomes can be seen, presumably via cross contamination and inherent tissue susceptibility [13].

There is an absence of scientific data by which to establish clear guidelines for the management of these types of patients. Recognizing that viral shedding could be intermittent, we empirically performed genital HSV cultures. After confirming a negative culture, we strategized that subsequent management would rely on the absence of typical patient symptomatology for recurrent genital disease. Although we gave consideration to planning an elective operative delivery to avoid any risk of vertical transmission to the neonate, this was determined to be counterintuitive, as we would be operating across abdominal skin with previously confirmed viral infection. We instead focused our strategies on treating and suppressing active HSV infection, to ultimately minimize the risk of transmission during attempted normal vaginal delivery.

The above reported therapeutic interventions for psoriasis appear to be safe in pregnancy and were of critical importance to achieve relative quiescence of the disease process [14]. Antiviral therapy, and later suppression, was a vital component for both maternal and neonatal outcomes. The initiation of adalimumab, a biologic TNF-alpha inhibitor, was strongly considered for gaining better control of the patient's psoriasis. In preclinical animal studies, this agent did not appear to show any adverse pregnancy-related effects [15]. An ongoing prospective adalimumab pregnancy registry has likewise been relatively reassuring concerning toxicities during the treatment of a variety of autoimmune disorders in pregnancy [16]. Ultimately for our patient, adalimumab therapy was deferred, given that the patient responded well to conservative therapy, as well as the uncertainty of additional immunosuppression in the setting of her HSV infection.

Despite the patient presenting with term rupture of membranes on active viral suppressive therapy with no genital symptomatology, we approached the delivery plan with trepidation. Although we ultimately proceeded with vaginal delivery with no specific surveillance or treatment of the neonate, we feel there is a strong argument for empirically culturing the neonate for HSV and possibly initiating short-term prophylactic antiviral therapy in this clinical scenario. In a case report describing an acute episode of maternal KVE during the intrapartum period, neonatal rectal cultures were positive for HSV [4]. Both mother and neonate were given parenteral acyclovir with favorable outcomes. This case differed from ours in that active HSV lesions were noted during labor, increasing the likelihood of vertical transmission.

The question of maternal isolation for a period of time from the neonate was also raised but not adopted as a strategy. This would have been a more pressing concern if the patient had active HSV lesions that could not be managed with barrier protection at the time of delivery. Further description of cases is needed to help better understand and optimally manage this rare and potentially devastating condition in pregnancy.

## References

[1] Santmyire-Rosenberger B. R., Nigra T. P. (2005). Psoriasis herpeticum: three cases of Kaposi's varicelliform eruption in psoriasis. *Journal of the American Academy of Dermatology*.

[2] Garg G., Thami G. P. (2012). Psoriasis herpeticum due to Varicella zoster virus: a Kaposi's varicelliform eruption in erythrodermic psoriasis. *Indian Journal of Dermatology*.

[3] George M., Pakran J., Rajan U., George S., Thomas S. (2011). Localized psoriasis herpeticum: case report and review of literature. *Indian Dermatology Online Journal*.

[4] DiCarlo A., Amon E., Gardner M., Barr S., Ott K. (2008). Eczema herpeticum in pregnancy and neonatal herpes infection. *Obstetrics and Gynecology*.

[5] Gurvits G. E., Nord J. A. (2011). Eczema herpeticum in pregnancy. *Dermatology Reports*.

[6] Latta R. A., Baker D. A. (1996). Treatment of recurrent eczema herpeticum in pregnancy with acyclovir. *Infectious Diseases in Obstetrics and Gynecology*.

[7] Miller O. B., Arbesman C., Baer R. L. (1950). Disseminated cutaneous herpes simplex (Kaposi's Varicelliform Eruption). *Archives of Dermatology and Syphilology*.

[8] Rekant S. I. (1973). Eczema herpeticum in pregnancy. *Obstetrics & Gynecology*.

[9] Garland S. M., Hill P. J. (1994). Eczema herpeticum in pregnancy successfully treated with acyclovir. *Australian and New Zealand Journal of Obstetrics and Gynaecology*.

[10] Wald A., Corey L. (2007). Persistence in the population: epidemiology, transmission. *Human Herpesviruses: Biology, Therapy, and Immunoprophylaxis*.

[11] Xu F., Sternberg M. R., Kottiri B. J. (2006). Trends in herpes simplex virus type 1 and type 2 seroprevalence in the United States. *The Journal of the American Medical Association*.

[12] Centers for Disease Control and Prevention (CDC) (2010). Seroprevalence of herpes simplex virus type 2 among persons aged 14–49 years—United States, 2005–2008. *Morbidity and Mortality Weekly Report*.

[13] Kimberlin D. W., Rouse D. J. (2004). Clinical practice: genital herpes. *The New England Journal of Medicine*.

[14] Pasternak B., Hviid A. (2010). Use of acyclovir, valacyclovir, and famciclovir in the first trimester of pregnancy and the risk of birth defects. *The Journal of the American Medical Association*.

[15] Mervic L. (2014). Management of moderate to severe plaque psoriasis in pregnancy and lactation in the Era of biologics. *Acta Dermatovenerologica Alpina, Pannonica et Adriatica*.

[16] Johnson D. L., Jones K. L., Chambers C. D., Salas E. (2009). 142 Pregnancy outcomes in women exposed to adalimumab: the OTIS autoimmune diseases in pregnancy project. *Gastroenterology*.

